# Sleep and mood disorders in dry eye disease and allied irritating ocular diseases

**DOI:** 10.1038/srep22480

**Published:** 2016-03-01

**Authors:** Masahiko Ayaki, Motoko Kawashima, Kazuno Negishi, Taishiro Kishimoto, Masaru Mimura, Kazuo Tsubota

**Affiliations:** 1 Department of Ophthalmology Keio University, School of Medicine, Tokyo, Japan; 2 Department of Psychiatry, Keio University, School of Medicine, Tokyo, Japan; 3 Department of Shinseikai Hospital Eye Center, Imizu, Japan

## Abstract

The aim of the present study was to evaluate sleep and mood disorders in patients with irritating ocular diseases. The study design was a cross-sectional/case-control study conducted in six eye clinics. Out of 715 outpatients diagnosed with irritating ocular surface diseases and initially enrolled, 301 patients with dry eye disease (DED) and 202 age-matched control participants with other ocular surface diseases were analyzed. The mean Pittsburgh Sleep Quality Index (PSQI) and Hospital Anxiety and Depression Scale (HADS) scores were 6.4 ± 3.2 and 11.1 ± 5.7 for severe DED (n = 146), 5.5 ± 3.3 and 9.8 ± 4.0 for mild DED (n = 155), 5.5 ± 3.1 and 9.5 ± 6.6 for chronic conjunctivitis (n = 124), and 5.0 ± 3.3 and 8.9 ± 5.3 for allergic conjunctivitis (n = 78). There were significant differences among these diagnostic groups for PSQI (*P *< 0.05). Regression analysis of patients with DED revealed the PSQI and HADS scores were significantly correlated with the severity of DED (*P *< 0.05). Our results demonstrate that sleep quality in patients with DED is significantly worse than in patients with other irritating ocular surface diseases and it is correlated with the severity of DED.

Dry eye disease (DED), chronic conjunctivitis, and allergic conjunctivitis are very common irritating eye diseases with symptoms including pain, burning, foreign body sensation, discomfort, discharge, and blurred vision, as well as dryness in DED and itching in allergic conjunctivitis. The lesions in ocular surface diseases are generally easy to recognize, however, DED has an unknown etiology and the reported signs and symptoms often have discrepancies^1^,^2^,^3^. Lubricant, steroid, immunosuppressant, or mucin secretagogue eyedrops are indicated for DED and even with treatment, patients often feel discomfort when there are exacerbating factors liable to worsen the ocular condition^4^. This is another reason why DED may be associated with depression and a low quality of life (QOL), resulting in a huge loss of productivity in the active Japanese working population^5^. We previously assessed sleep and depression in ophthalmic outpatients undergoing routine ophthalmic examinations^6^ and found patients with DED suffered more seriously from sleep and mood problems than patients with other eye diseases such as glaucoma or cataract. A recent investigation also indicated the symptoms of DED were closely correlated with subjective happiness^7^, suggesting patients with DED may have a considerable psychiatric profile.

To the best of our knowledge, no study has reported whether any ocular surface disease induces sleep disorders, although every ocular surface disease may produce symptoms that may induce distress, potentially leading to sleep disorders. Ocular pain and discomfort are generally mediated by the trigeminal nerve and the discomfort spreads widely and radiates around the eyes. We hypothesized that if depression is caused by ocular surface discomfort, patients with any ocular surface disease might accordingly develop depression and sleep disorders.

In this study, we conducted a comprehensive ophthalmological examination along with a questionnaire-based survey, which included questions about sleep, depression, anxiety, morningness/eveningness, and photophobia, in patients with ocular irritating diseases to evaluate their sleep and mood status. We compared psychiatric indices among patients with DED and allied irritating ocular surface diseases.

## Methods

### Study institutions and Institutional Review Board approval

Participants were recruited to the study from eye clinic patients at six institutions in Japan between January 2014 and December 2014. The Institutional Review Board and Ethics Committee of the Keio University School of Medicine approved this study and the methods were carried out in accordance with the Declaration of Helsinki. Informed consent was obtained from all participants.

### Questionnaire-based survey

Patients were invited to fill out questionnaires, which included the Pittsburgh Sleep Quality Index (PSQI)^8^,^9^ and the Hospital Anxiety and Depression Scale (HADS)^10^,^11^. Each questionnaire was self-administered during the patient visit and performed from January through March, 2014. The score for each scale was calculated according to separate algorithms and the scores were then subjected to analysis. The cut-off points for possible sleep and mood disorders were 5/6 for PSQI^8^ and 9/10 for HADS^10^. PSQI is composed of seven subscales and HADS is composed of depression (HADS-D) and anxiety (HADS-A) subscores. These questionnaires have been widely used for hospital-based surveys and are easy to answer because they do not contain questions concerning severe psychiatric disease (eg suicide and hallucination). Photophobia and chronotype (morningness/eveningness) were evaluated with two representative questions from established questionnaires (National Eye Institute Visual Function Questionnaire-25^12^ and Morningness/Eveningness questionnaire^13^). The photophobia score ranged from 100 (best) to 0 (worst) and the morningness/eveningness score ranged from 10 (far morningness) to 0 (far eveningness). We measured the severity of photophobia since it is one of the major symptoms of DED^14^ and it may be associated with sleep and mood status.

### Ophthalmological examination

All outpatients were examined by board-certified ophthalmologists specialized in corneal disorders and certified orthoptists, and their diagnosis of ocular surface disease was classified as DED, allergic conjunctivitis, or chronic conjunctivitis. A diagnosis of DED was made according to the Japanese Dry Eye Society^15^,^16^, which classifies DED into definite DED (DDED), probable DED (PDED), and non DED according to the presence of DE symptoms, tear abnormality (Schirmer test ≤ 5 mm or tear break-up time [BUT] ≤ 5 sec), and corneoconjunctival epithelial damage (staining score ≥ 3). Patients diagnosed with DDED or PDED were enrolled as DED and any patient using eyedrops other than hyaluronate, mucin secretagogue, or steroid was excluded. The severity of DED was determined as mild DED if symptoms and signs were controlled by hyaluronate only. The patients with symptoms of dryness and ocular pain that were not controlled with hyaluronate and/or with DED signs including persistent epithelial defects and conjunctival injection were prescribed additional medications and classified as severe DED in this study. Mucin secretagogue and/or steroid eyedrops were prescribed for patients with severe symptoms and/or a short BUT at their first visit. No patient had non-medical interventions, including punctal plug insertion, punctal occlusion, or other surgical interventions.

Allergic conjunctivitis was diagnosed with symptoms and signs including itching, irritation, injection, lacrimation, and eye discharge corresponding to conjunctival edema, papillae, follicles, or injection. Patients in the allergic conjunctivitis group were seen at a returning visit and/or they had a typical history of seasonal or perennial allergic conjunctivitis clearly responsive to histamine blocker and/or steroid eyedrops. Chronic conjunctivitis was diagnosed with eye discharge, irritation, pain, or any other discomfort without corneoconjunctival epithelial disorders. Patients in the chronic conjunctivitis group were seen at regular visits or they had symptoms lasting for more than a month.

The prescribed eye drops for the treatment of DED were hyaluronate, mucin secretagogue, and steroid, i.e., 0.1%/0.3% Hyalein^R^ (sodium hyaluronate; Santen Pharmaceutical Co. Ltd., Osaka, Japan), 0.1%/0.3% Tearbalance^R^ (sodium hyaluronate; Senju Pharmaceutical Co. Ltd., Osaka, Japan), Diquas^R^ (3% diquafosol sodium; Santen Pharmaceutical Co. Ltd), Mucosta^R^ (2% rebamipide, Otsuka Pharmaceutical, Co. Ltd., Tokyo, Japan), and 0.02%/0.1% Flumetholon^R^ (fluorometholon; Santen Pharmaceutical Co. Ltd). We prescribed various histamine blockers or steroid eyedrops for allergic conjunctivitis; antibiotics, steroids, and/or nonsteroidal anti-inflammatory eyedrops for chronic conjunctivitis; and hyaluronate for trichiasis (included in the chronic conjunctivitis group) according to the symptoms of the patient.

### Exclusion criteria

Patients were excluded if they also had severe glaucoma (mean deviation* *< −12 dB in either eye), bilateral cataracts, visual impairment (<20/30 in both eyes), acute disease presenting for less than one month, or were* *< 20 years old. Age was further matched among the diagnostic groups since many patients with allergic conjunctivitis were* *< 30 years old.

### Statistical analysis

Where appropriate, data are given as the mean ± standard deviation. The mean score and proportion of patients with high values for the HADS and PSQI scores in each diagnostic group were calculated and compared among diagnostic groups. To identify which ophthalmic parameters were correlated with psychiatric problems (sleep and mood disorders) in DED, a regression analysis of patients with DED was carried out, with PSQI and HADS scores as dependent variables and demographic (gender and age) and ophthalmic parameters as independent variables. All analyses were performed using StatFlex (Atech, Osaka, Japan) and SPSS version 21 (SPSS Inc., Chicago, IL), with *P *< 0.05 considered significant.

## Results

A total of 715 outpatients initially participated in this study and finally 301 patients with DED and 202 age-matched control participants with other ocular surface diseases were analyzed following inclusion and exclusion criteria. Patient demographics and ocular parameters are summarized in Table 1. Tear BUT was significantly different between patients with mild and severe DED (*P *< 0.05, unpaired *t* test). The female gender was predominant in DED and allergic conjunctivitis. There were no differences between diagnostic groups for the other parameters of age, myopic errors, and phakic status. Eyedrop prescriptions for ocular surface diseases were consistent with diagnosis.

**Figure 1:**
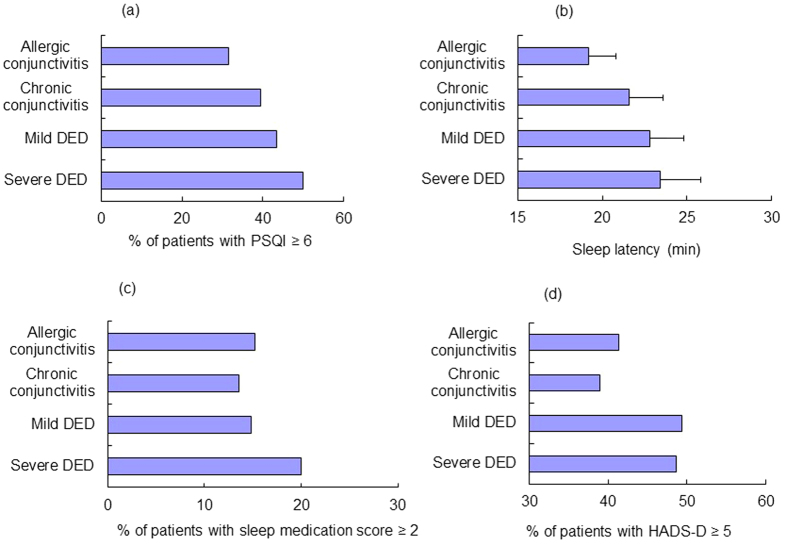
(**a**) Proportion of patients with a Pittsburgh Sleep Quality Index (PSQI) global score ≥ 6; (**b**) sleep latency time; (**c**) proportion of patients with a sleep medication score ≥ 2; and (**d**) proportion of patients with a Hospital Anxiety and Depression Scale depressive subscore ≥ 5 in the four diagnostic groups. Generally, sleep quality was worst in patients with severe dry eye disease (DED) followed by patients with mild DED. The sleep medication score (3 = taking sleep medication more than three days a week, 2 = one or two days a week, 1 = less than once a week, 0 = none) was highest in patients with severe DED.

**Figure 2:**
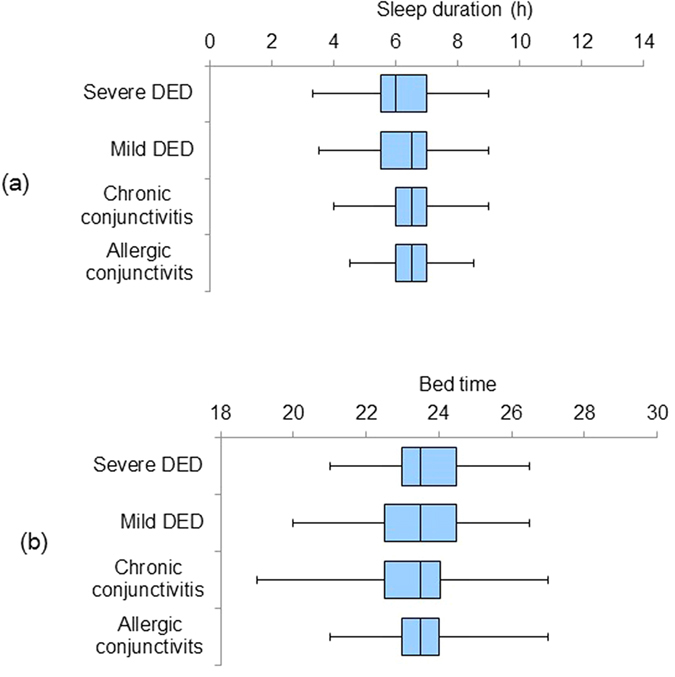
Box plots of distributions of (**a**) sleep duration; and (**b**) bed time in the four diagnostic groups. Patients with severe dry eye disease (DED) went to bed latest (n.s., Kruskal-Wallis test) and their sleep duration was the shortest (*P *< 0.05). The vertical line in each diagram indicates the median. The width, positive error bar, and negative error bar of each box indicate the 25%/75% percentile, maximum value, and minimum value, respectively.

**Figure 3:**
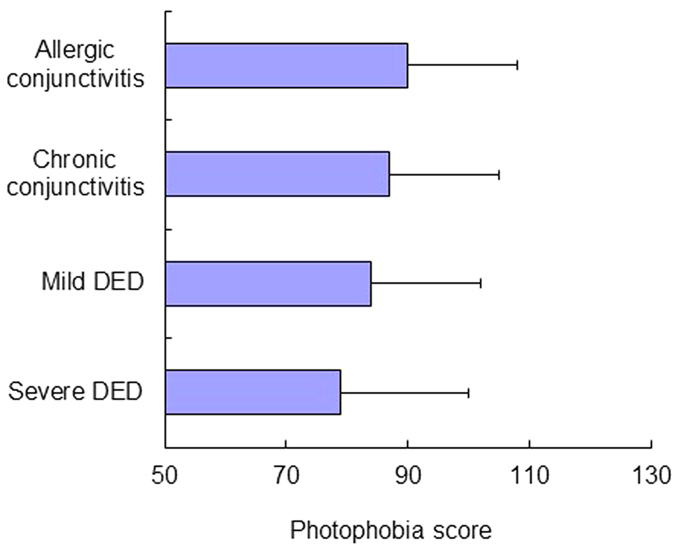
Photophobia scores for the four diagnostic groups. The values (100 = best, 0 = worst) followed the same pattern as for the sleep quality indices (Fig. 1a,b). There are significant differences among the four diagnostic groups (*P *< 0.001, Kruskal-Wallis test).

Patients with severe DED had the worst score for PSQI (*P *< 0.05, Kruskal-Wallis test), sleep duration (*P *< 0.05), sleep efficacy (*P *< 0.05), and HADS (*P* = 0.07) (Table 2). The proportion of patients with poor scores and the sleep latency of patients are shown in Fig. 1. The distribution of values of sleep duration and bed time are expressed in box plots (Fig. 2). Generally, the severe DED group had the poorest scores and the mild DED group had the second poorest scores for sleep and mood indices. Additionally, photophobia was significantly worse in the severe DED group, compared to all the other groups (*P *< 0.05, Kruskal-Wallis test; Fig. 3), while the levels of photophobia in the diagnostic groups corresponded to their global PSQI scores. The morningness/eveningness score was 4.5 ± 2.3 for severe DED, 4.7 ± 2.3 for mild DED, 4.6 ± 2.2 for chronic conjunctivitis, and 4.4 ± 2.3 for allergic conjunctivitis, with no significant difference between the diagnostic groups.

Stepwise regression analysis of the DED groups revealed that the PSQI score was significantly correlated with mood indices (*P *< 0.001, Pearson correlation coefficient), the severity of DED (*P *< 0.05), photophobia (*P *< 0.001), and morningness/eveningness (*P *< 0.001). The HADS score in patients with DED was significantly correlated with the severity of DED (*P *< 0.05), photophobia (*P *< 0.001), and morningness/eveningness (*P *< 0.05; Table 3).

## Discussion

The present results demonstrated poor sleep quality in patients with DED. This finding was comparable with our previous survey results where the DED group included many patients with overlapping ocular diseases^6^. However, the results of the chronic and allergic conjunctivitis groups were worse than those in the previous report as many young patients were excluded in the current study to ensure age-matching of the DED groups. The present results implicate sleep disorders as one of the contributing factors for low QOL in patients with DED, since sleep is critical for health and survival^17^,^18^,^19^.

**Figure 4:**
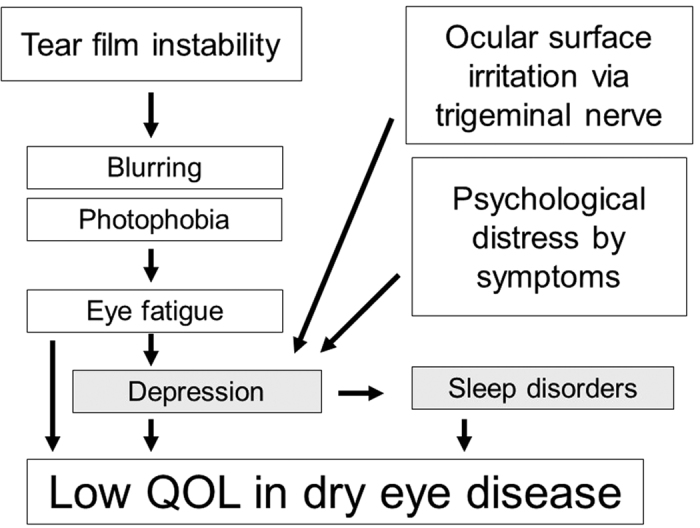
Hypothesis of sleep disorders and low quality of life (QOL) in patients with dry eye disease. Patients may be distressed by sensory discomfort, optical disturbance, or by psychological distress from the disease itself. These factors affect patients and may lead to depression, sleep disorders, and low QOL.

Considerably disturbed sleep quality in severe DED was correlated with mood and not with the clinical parameters of DED, including the Schirmer test, tear BUT, and keratoepitheliopathy. We speculated that a psychiatric factor, namely, distress due to DED, could be a major possible cause of such sleep disorders. Participating ophthalmologists determined the prescription of eyedrops mostly by the severity of symptoms, especially in cases with mild signs. Eventually patients with severe DED had steroid and/or mucin secretagogue eyedrops for their severe symptoms, which were presumably causing distress. Optical disability and eye fatigue may also cause distress for DED patients (Fig. 4). Eye fatigue is a common symptom of DED, presumably due to photophobia and blurred vision induced by tear film instability^20^.

Patients with severe DED went to bed late, slept less, and had more sleep medication compared to the other diagnostic groups. These are common features in poor sleepers and may not suggest DED-specific conditions. Some patients with DED may have taken sleep medication containing anticholinergic agents, which suppress tear secretion^21^,^22^; however, this scenario was unlikely in the present study since only approximately 20% of patients with DED regularly took sleep medication (Fig. 1c). Alternatively, we speculate that patients with DED developed a depressive disorder^23^,^24^,^25^,^26^ and, at the same time, a sleep disorder, due to distress from DED. Long-term use of antidepressants may also be a potential cause of DED^27^. Whether depression or sleep disorder is a cause or result of DED should be definitely further examined.

Even though most ocular surface diseases result in the same physiological phenomena of somatic pain and discomfort, DED had the worst PSQI and HADS scores. What makes DED different to other ocular diseases? Patients with DED suffer from continuous irritation due to dryness and in severe DED, the symptoms continue for life and can worsen at any time. Therefore, patients with DED may be frequently anxious about their eyes. Consequently, DED can cause a constant feeling of discomfort or distress that leads to mood disorders. On the other hand, patients with chronic and allergic conjunctivitis may clearly recognize the cause of their irritation and expect to be adequately treated.

Photophobia is a complicated manifestation associated with sensitivity to light, blurred vision, and deep ocular pain^28^,^29^. It is a common symptom of DED and possibly comes from the uneven ocular surface caused by the unstable tear film. Glaucoma and cataract are eye diseases with pathology that could potentially lead to sleep disorders, since intrinsically photosensitive retinal ganglion cells are damaged in glaucoma causing deterioration of circadian photoreception^30^, and cataract blocks blue light irradiation to the retinal ganglion cells^31^. We therefore excluded any patients with glaucoma or cataract from the present study. DED is a multifactorial disease with ocular surface abnormalities in the tear film and corneoconjunctival epithelium; however, lacrimal secretion is controlled by the central nervous system. Systemic, neural, and psychiatric complications in DED need to be further clarified.

### Limitations

Limitations of this study are the self-administered questionnaire-based nature of evaluating sleep and the lack of systemic evaluation of the participants. Sleep quality should be further examined with polysomnography and actigraphy. Severity of DED was mostly based on the clinical decisions of the participating ophthalmologists and should be made according to established criteria. Actually, there were no significant differences in ocular and psychiatric parameters between PDED and DDED, except for corneoconjunctival epitheliopathy (data not shown), which is why we showed significant results under the classifications of severe and mild DED. There is also the potential for the overlapping of DED and other diseases, and a small number of patients with DED may have been included in the allergy and chronic conjunctivitis groups. This study should be further confirmed using a comprehensive DED classification, such as the one proposed by the 2007 Dry Eye Workshop^32^.

## Conclusions

In conclusion, the present results indicate patients with DED suffered more seriously from sleep and mood problems compared to patients with other irritating ocular surface diseases. We speculate that sleep and mood disorders in these patients could derive from a disease-specific ocular surface abnormality and optical disability. Psychiatric treatment focusing on sleep and mood disorders might therefore be beneficial for patients with DED.

## Additional Information

**How to cite this article**: Ayaki, M. *et al.* Sleep and mood disorders in dry eye disease and allied irritating ocular diseases. *Sci. Rep.* **6**, 22480; doi: 10.1038/srep22480 (2016).

## Figures and Tables

**Table 1:** Patient demographics.

Diagnostic group	Severe DED	Mild DED	All cases of DED	Chronic conjunctivitis	Allergic conjunctivitis
No. of patients	146	155	301	124	78
% Male	16.9	16.2	16.5	42.4	21.3
Age (y)	61.7 ± 14.4	60.2 ± 16.5	61.0 ± 15.5	61.0 ± 16.8	58.2 ± 15.1
Median (y)	64	63	63	63	60
Range (y)	22–89	20–87	20–89	24–91	31–87
High myopia (≤ −6.00D) (%)	10.0	8.4	9.2	7.1	13.8
Bilateral IOL (%)	10.0	9.6	9.8	7.9	10.0
Clinical parameters of ocular symptoms					
Definite DED (%)	29.3	24.4	26.9		
Epitheliopathy (%)	69.4	68.3	68.9		
Schirmer test (mm)	12.6 ± 6.2	11.4 ± 5.6	12.0 ± 5.9		
Tear BUT (s)	3.5 ± 2.5	4.6 ± 2.9*	4.0 ± 2.7		
Topical medication (%)					
Steroid	31.3	0	14.9	41.7	37.5
NSAID/Histamine blocker	0	0	0	14.2	75.0
Hyaluronate	58.7	100	80.3	6.3	0
Mucin secretagogue	69.3	0	32.9	0	0
Antibiotic	0	0	0	33.9	0

Data are mean ± standard deviation unless otherwise indicated. ^*^P < 0.05, severe vs. mild DED. Abbreviations: DED, dry eye disease; BUT, tear break-up time; IOL, intra-ocular lens, NSAID, nonsteroidal anti-inflammatory drug.

**Table 2:** Diagnostic groups and representative psychiatric results.

Diagnostic group	Sleep indices				Mood indices		
	PSQI score*	Sleep duration*	Sleep efficacy*	Sleep medication	HADS score	HADS-A subscore	HADS-D subscore
Severe DED	6.4 ± 3.3	1.5 ± 0.8	0.40 ± 0.77	0.56 ± 1.09	11.1 ± 6.6	5.4 ± 3.6	5.3 ± 3.7
Mild DED	5.5 ± 3.1	1.3 ± 0.8	0.29 ± 0.66	0.42 ± 1.01	9.8 ± 5.7	5.2 ± 3.1	4.6 ± 3.4
Chronic conjunctivitis	5.5 ± 3.2	1.3 ± 0.9	0.32 ± 0.70	0.43 ± 0.96	9.5 ± 5.6	4.1 ± 3.1	4.5 ± 3.3
Allergic conjunctivitis	5.0 ± 2.8	1.3 ± 0.8	0.23 ± 0.58	0.43 ± 0.90	8.9 ± 6.0	5.0 ± 3.4	4.2 ± 3.3

Data are mean ± standard deviation. ^*^P < 0.05, Kruskal-Wallis test. Abbreviations: DED, dry eye disease; PSQI, Pittsburgh Sleep Quality Index; HADS, Hospital Anxiety and Depression Scale; HADS-A, anxiety subscore of HADS; HADS-D, depression subscore of HADS.

**Table 3:** Regression analysis of psychiatric indices and demographic and ophthalmic parameters in 301 patients with dry eye disease (DED).

	PSQI			HADS	
	β	*P* value		β	*P* value
Age	0.126	0.03		−0.075	n.s.
Gender^A^	0.061	n.s.		0.034	n.s.
Mood index					
HADS	0.420		< 0.001	–	–
HADS-A	0.370		< 0.001	–	–
HADS-D	0.388		< 0.001	–	–
Symptoms and signs					
High myopia	0.062		n.s.	0.057	n.s.
Photophobia^B^	−0.237		< 0.001	−0.299	< 0.001
Morningness/eveningness^C^	−0.225		< 0.001	−0.121	< 0.05
Severity of DED^D^	0.120		< 0.05	0.119	< 0.05
BUT	−0.062		n.s.	−0.031	n.s.
Schirmer test	−0.079		n.s.	−0.216	n.s. (0.07)
Keratoepitheliopathy	0.054		n.s.	−0.016	n.s.
Medication for DED					
Hyaluronate	0.051		n.s.	−0.051	n.s.
Mucin secretagogue	0.151		n.s. (0.08)	0.133	n.s.
Steroid	0.081		n.s.	−0.003	n.s.

Pearson product moment correlation. ^A^Male = 1, female = 0. ^B,C,D^See text in methods. Abbreviations: PSQI, Pittsburgh Sleep Quality Index; HADS, Hospital Anxiety and Depression Scale; HADS-A, anxiety subscore of HADS; HADS-D, depression subscore of HADS; BUT, tear break-up time; n.s., not significant.
